# YOLO12 Down Feather Quality Classification Method Based on A2C2f-CGLU Feature Enhancement

**DOI:** 10.3390/s26185826

**Published:** 2026-09-14

**Authors:** Zhihui Fan, Shaowen Jing, Lihong Tong, Xihong Sun

**Affiliations:** School of Information Engineering, Henan University of Science and Technology, Luoyang 471023, China

**Keywords:** down feather quality classification, object detection, YOLO12, A2C2f-CGLU, Convolutional Gated Linear Unit, fine-grained recognition

## Abstract

Manual sorting is still the mainstream scheme for component identification in down feather quality evaluation, which suffers from low detection efficiency and poor stability. To address these drawbacks, this paper proposes a fine-grained component detection algorithm based on YOLO12 for down feather quality classification. Five typical down feather components are selected as detection targets, including discolored feathers, down filaments, immature down, feathers and pure down. A dedicated down feather object detection dataset consisting of 1140 images is established accordingly. To improve the feature representation capacity of the model for tiny objects, faint boundary features and subtle distinctions between analogous categories, this work integrates the Convolutional Gated Linear Unit (CGLU) into the A2C2f module of YOLO12. While maintaining the original feature aggregation pathways and residual architecture, the conventional MLP feed-forward branch within ABlock is replaced with convolutional gated transformation. Experimental results demonstrate that the proposed A2C2f-CGLU model achieves precision of 96.50%, recall of 94.49%, mAP50 of 98.05% and mAP50-95 of 57.89% with the optimal weights on the validation set. Compared with the original YOLO12, the mAP50-95 metric is elevated by 3.04 percentage points, and the overall performance surpasses two comparative variants, A2C2f-DFFN and A2C2f-KAN. Visualizations of PR curves, confusion matrices and real test samples validate that the proposed method effectively enhances the recognition stability of tiny down feather targets and similar classes. This research provides a visual inspection foundation for subsequent component proportion calculation, quality grade discrimination and the development of intelligent detection systems.

## 1. Introduction

Down is a kind of natural animal fiber with excellent loftiness, thermal insulation and lightweight properties, which is widely adopted in garments, bedding, outdoor thermal supplies and other products. The quality of down products not only determines their thermal retention performance and wearing comfort, but also directly affects product grading and market value. In practical production and quality inspection, down samples are generally mixtures of multiple components including pure down, immature down, down filaments, feathers and discolored feathers (predominantly yellow feathers). The category and proportional content of each component serve as critical criteria for evaluating down quality. Accordingly, accurate identification of fine-grained components within down samples, which can furnish credible data support for subsequent quality classification and assessment, lays an essential foundation for realizing automatic, standardized and intelligent down inspection

At present, the quality inspection of down still largely relies on manual sorting, weighing and statistics. Inspectors need to separate and identify various down components one by one with tweezers, glass platforms or microscopic observation equipment. Despite its intuitiveness, this manual inspection scheme suffers from low efficiency, heavy labor intensity, strong subjective bias and poor repeatability. Especially for mass production inspection scenarios, manual detection fails to simultaneously satisfy the requirements of high speed, stable performance and traceability. Disparities in professional experience among different inspectors also easily cause fluctuations in detection results. As the down industry evolves toward large-scale, refined and intelligent manufacturing, conventional manual inspection methods can no longer meet the demands of high-efficiency and high-consistency quality testing.

The core of down feather quality classification lies not merely in determining whether a specific component exists in samples, but in stably acquiring the category, position and quantity information of each component. In practical inspection scenarios, a single down feather image usually contains multiple targets with similar shapes, ambiguous boundaries and drastically varying scales, and the detection outputs directly govern the subsequent calculation of component proportions and quality grading. For this reason, this paper formulates down feather image analysis as a fine-grained visual detection task oriented to quality evaluation, rather than a conventional single-object recognition task. In recent years, computer vision and deep learning approaches have been extensively applied to quality assessment tasks such as food safety inspection, livestock and poultry product detection, and surface defect identification of industrial goods. Relevant studies have verified that deep object detection models can learn morphological contours, local textures and spatial distribution features of targets during end-to-end training, establishing a technical foundation for the automatic identification and objective evaluation of complex samples [1,2,3,4]. Among mainstream detection architectures, the YOLO series delivers a favorable trade-off between detection accuracy, inference speed and deployment convenience owing to its one-stage pipeline, rendering it one of the representative frameworks for real-time object detection. In recent research, YOLO and its improved variants have been adopted for industrial visual inspection tasks including textile defects, product surface flaws, leather blemishes and electronic component defects, which demonstrates its engineering practicability for the rapid detection of mass samples [5,6,7,8,9,10,11,12,13,14]. On the above basis, the YOLO framework is selected as the baseline for down feather component detection in this work. Targeted structural modifications are further proposed to accommodate the inherent characteristics of down samples, including loosely shaped targets, wide scale variation and high inter-class similarity.

Although existing object detection models can realize basic recognition of down feather components, they still suffer from insufficient feature representation when processing complex down samples. Down feather targets are characterized by strong morphological flexibility, ambiguous boundaries, random postures and high inter-class similarity. Distinctions between different categories mainly lie in local fiber structures, edge branching shapes and subtle texture variations. If such local discriminative information cannot be fully preserved during feature extraction and multi-scale feature fusion, the model tends to confuse analogous components, thereby impairing the reliability of quality evaluation results. Previous studies on small-object detection have proven that tiny target sizes, limited distinguishable regions, severe background interference and dense target distribution degrade the model’s capacity to capture detailed features and raise the risks of missed detection and false detection [15]. For visual inspection tasks involving fabrics and nonwovens with fine textures and local defect features, the relevant literature has demonstrated that strategies including strengthened local feature extraction, multi-scale fusion and attention screening mechanisms can boost the model’s discrimination ability for targets with subtle inter-category differences [16,17]. Moreover, improved YOLO variants designed for small and dense object detection further verify that context enhancement, lightweight multi-scale attention and local detail retention mechanisms can mitigate the degradation of small-object features in deep network layers [18,19,20]. In view of the prominent issues in down feather images, namely abundant tiny targets, faint local texture differences and indistinct category boundaries, it is necessary to embed more effective feature enhancement and local information screening modules into the baseline detection model.

To tackle the aforementioned limitations, this paper proposes a YOLO12 fine-grained component detection method with A2C2f-CGLU feature enhancement, which can deliver more reliable visual evidence for subsequent down feather quality classification. Taking YOLO12 as the baseline detection framework, the Convolutional Gated Linear Unit (CGLU) [21] is integrated inside the A2C2f module to construct the A2C2f-CGLU feature enhancement architecture. Unlike simply stacking extra modules, the proposed scheme retains the original feature aggregation pathways and residual structures of YOLO12, while replacing the vanilla MLP feed-forward branch within ABlock with convolutional gated transformation. This design enables the model to incorporate local convolutional perception and gated feature screening mechanisms during channel-wise interaction, so as to strengthen its representation capability for local textures, faint boundaries and subtle inter-class discrepancies of down feather targets.

In terms of model architecture, the proposed A2C2f-CGLU inherits the original regional attention modeling capability of A2C2f while further boosting local texture feature extraction and channel-wise feature selection. Without disrupting the backbone information flow of YOLO12, this design improves the model’s representational capacity for tiny objects, dense targets and targets with irregular boundaries. Therefore, the method is better adapted to the inspection scenarios of down feather images, which feature coexistence of multiple component categories, complex target morphologies and subtle inter-class discrepancies.

To verify the effectiveness of the proposed method, a fine-grained object detection dataset dedicated to the down feather quality classification task is constructed in this work, covering five types of down-related components as detection targets, namely yellow_feather, down_silk, immature_down, feather and down. On this basis, comparative experiments are carried out among four models: the vanilla YOLO12, A2C2f-DFFN [22], A2C2f-KAN [23] and the proposed A2C2f-CGLU. Comprehensive performance evaluations are conducted from multiple dimensions including precision, recall, mAP50, mAP50-95, PR curves, confusion matrices and visualization results of real samples, so as to validate the applicability of A2C2f-CGLU for fine-grained component detection of down feathers.

The main contributions of this work are summarized as follows:

(1) A fine-grained object detection dataset tailored for down feather quality classification is established. Annotated in YOLO format, this dataset covers five categories of down feather components, i.e., yellow_feather, down_silk, immature_down, feather and down. It can support localization, identification of key components in down samples as well as subsequent analysis for quality classification.
(2) An improved YOLO12 architecture embedded with A2C2f-CGLU is proposed. By integrating the CGLU into the A2C2f module, the proposed structure leverages local convolutional perception and gated feature screening mechanisms to strengthen the model’s representation capability for tiny textures, faint boundaries and subtle inter-class discrepancies of down feather targets.
(3) Comparative experiments targeting the fine-grained detection of down feather components are conducted. The proposed A2C2f-CGLU is compared with vanilla YOLO12, A2C2f-DFFN and A2C2f-KAN. Combined with overall detection metrics, class-wise PR curves, confusion matrices and visualization results of real samples, the detection performance and practical applicability of the presented method are comprehensively validated.

## 2. Materials and Methods

Focusing on the fine-grained detection task of down feather components, this section elaborates on the research materials, dataset construction pipeline, image preprocessing and data augmentation strategies, as well as the baseline YOLO12 object detection model. Firstly, the source of down feather samples, detection categories and fundamental composition of the dataset are introduced, clarifying the core components investigated in this study, including pure down, immature down, down filaments, feathers and discolored feathers. Secondly, multiple data augmentation strategies adopted during model training are illustrated, such as geometric transformation, color jittering, mosaic and local perturbation, which are adopted to boost the model’s adaptability to complex down samples, tiny objects, faint boundaries and densely distributed targets. Finally, the fundamental architecture of the YOLO12 detection framework is presented, laying a methodological foundation for the design of the A2C2f-CGLU feature enhancement module and subsequent experimental validation.

### 2.1. Materials

**Figure 1:**
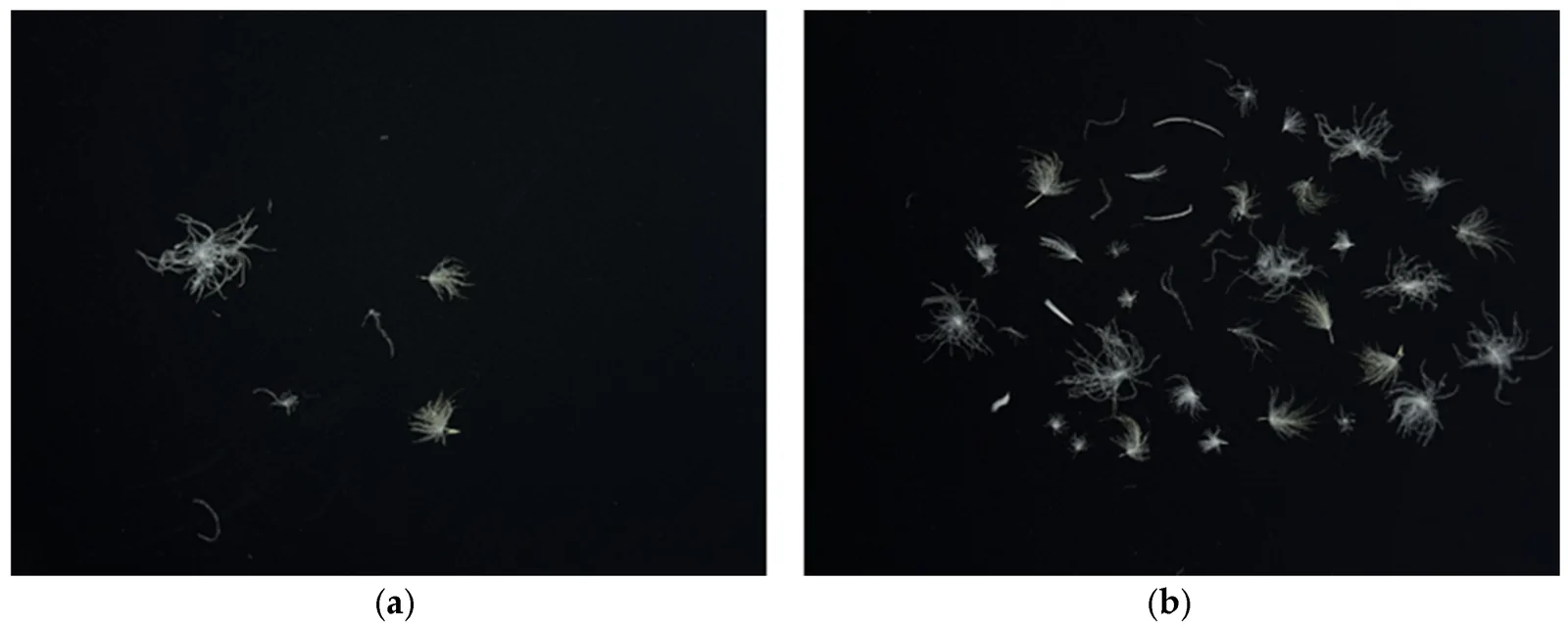
(**a**) Down feather samples with sparsely distributed targets. (**b**) Down feather samples with densely distributed multiple targets.

The research objects of this work are typical down feather specimens for down quality inspection, which are collected from a down processing factory in Taiqian County, Puyang City, Henan Province, China. To provide a reliable visual data basis for down feather quality classification, a custom object detection dataset for the fine-grained identification of down components is constructed, as illustrated in Figure 1a,b. Based on real-world images captured during industrial inspection, this dataset defines five detection categories: yellow_feather, down_silk, immature_down, feather and down, which correspond to discolored feathers, down filaments, immature down, feathers and pure down respectively. These components are the primary indicators closely associated with quality evaluation.

The dataset covers two types of samples: simple specimens with relatively sparsely distributed targets, and complex specimens featuring coexisting multiple targets, dramatic scale variations and adjacent object boundaries. Unlike idealized single-target images, real collected down samples generally exhibit random spreading, unfixed poses, ambiguous boundaries and partial overlapping regions. Consequently, the dataset highly approximates the image distribution under practical inspection environments and can be utilized to evaluate the model’s performance in tiny target localization, faint boundary retention and discrimination of multiple instances.

**Figure 2:**
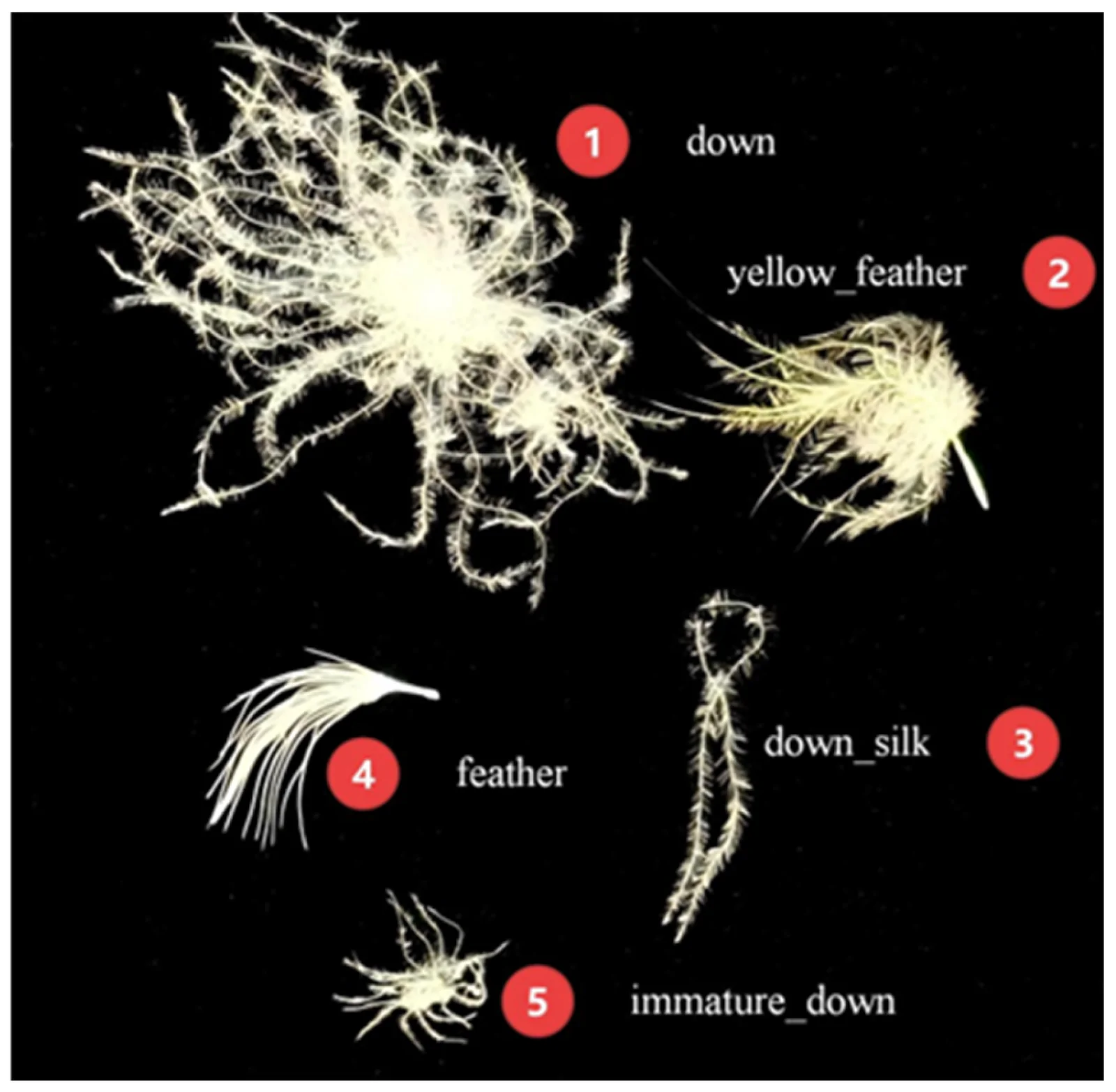
Schematic diagram of down feather categories.

As shown in Figure 2, this category diagram illustrates the visual discrepancies and labeling criteria for the five target categories: down, yellow_feather, down_silk, feather and immature_down. It can be observed that these down feather targets cannot be distinguished merely by color or size; their distinctions mainly lie in fine-grained features such as fiber branching, cluster compactness, rod-like structures and edge morphology. This figure demonstrates the reliance of down feather quality classification on fine-grained object detection from the data perspective, and further explains why the subsequent model requires enhanced local texture modeling and discriminative capacity for analogous categories.

### 2.2. Data Augmentation

To improve the model’s adaptability to complex down feather samples, online data augmentation is applied to input images during the training phase. First, all original images are resized to the unified input resolution of the model, and the bounding box information of targets is transformed synchronously following the YOLO annotation format to guarantee consistency between target positions and label information after augmentation. Image augmentation methods can expand the distribution of training samples and thereby boost the generalization performance of deep models. Common augmentation strategies include geometric transformation, color jittering, sample mixing, and local occlusion perturbation [24].

In terms of geometric transformations, several augmentation operations including random rotation, translation, scaling, shearing and horizontal flipping are adopted in this work. These operations expand the spatial distribution range of targets within images, enabling the model to adapt to variations in position, scale and orientation of down feather samples captured under practical acquisition conditions. Given the irregular shapes and random placement angles of down feather targets, geometric augmentation improves the model’s robustness against pose variations and the local deformation of objects. For color space augmentation, random perturbations are applied to the hue, saturation and brightness of images, simulating the influences of varying illumination, shooting environments and color differences of samples on imaging results. Existing studies on object detection have verified that geometric augmentation and light disturbance can enhance the generalization ability of detection models under complex data acquisition scenarios [25].

In terms of sample composition augmentation, Mosaic and MixUp strategies are utilized to fuse multiple images or information from distinct samples, thereby diversifying the quantity, scale and spatial arrangement of targets in each image. Such augmentation methods enrich background combinations and co-occurrence patterns of targets in training samples, which fits the detection scenarios of down feather images featuring dense tiny objects and coexisting multi-class targets. Relevant research has demonstrated that Mosaic-based data augmentation can effectively increase sample complexity and boost the detection performance of object detection models under multi-target and complex background conditions [26].

In addition, random erasing and auto-augment strategies are introduced to impose random perturbations on local image regions and overall augmentation pipelines, further enhancing the model’s adaptability to occlusion, partial feature loss and complex background interference. Research on automated image augmentation has proven that searching or learning augmentation policies can strengthen the robustness and generalization performance of models across various visual tasks [27]. Mixed precision training and cosine learning rate scheduling are adopted throughout the training process to accelerate training efficiency and optimize model convergence. Overall, the data preprocessing and augmentation strategies proposed in this work expand the distribution of training samples from four dimensions: geometric deformation, color perturbation, sample blending and local disturbance. These strategies provide richer training conditions for the YOLO12-A2C2f-CGLU model to learn fine-grained textures and faint boundary features of down feather targets.

### 2.3. YOLO12

YOLO12 serves as the baseline object detection framework adopted in this paper. Following the end-to-end detection paradigm of the YOLO family, its overall architecture mainly consists of three components: a backbone network for feature extraction, a multi-scale feature fusion module, and detection heads. After the input image is fed into the backbone to extract multi-level visual features, the model enhances the feature representation of objects at diverse scales via multi-scale feature fusion. Finally, the detection heads execute object category prediction and bounding box regression.

For the fine-grained detection task of down feather components, the prominent advantage of YOLO12 lies in its capability to simultaneously localize and classify multiple objects while maintaining high inference efficiency. This characteristic fits the practical inspection demands of down feather production, including coexistence of various components, abundant tiny targets, and high throughput requirements for batch image processing. Nevertheless, the vanilla YOLO12 still suffers from inherent limitations when processing down feather images. Down feather targets generally feature ambiguous boundaries, strong morphological flexibility, intricate local textures and subtle inter-class differences. If the model fails to fully retain local texture and faint boundary information during feature extraction, it tends to misclassify analogous categories or miss tiny targets.

Accordingly, targeted modifications are implemented on the vanilla YOLO12 architecture, focusing on boosting the local texture modeling capacity of the feed-forward branch inside the A2C2f module. The construction pipeline of the A2C2f-CGLU feature enhancement structure and its internal mechanism for fine-grained down feather object detection will be elaborated in the following sections.

## 3. Improvements to YOLO12

To strengthen the feature representation capability of YOLO12 for the fine-grained detection of down feather components, an A2C2f-CGLU feature enhancement architecture is integrated into the vanilla YOLO12 network. This modification preserves the overall pipeline of YOLO12, namely backbone feature extraction, neck multi-scale fusion and detection head prediction, while only replacing the feed-forward branch of ABlock inside the A2C2f module. Specifically, the Convolutional Gated Linear Unit (CGLU) is embedded into ABlock to reconstruct the original vanilla MLP feed-forward branch into a convolutional gated transformation. As a result, the model retains the regional attention modeling capability and achieves improved representation for local textures, faint boundaries and subtle inter-class discrepancies of down feather targets.

Distinct from simply stacking extra modules, the optimizations of A2C2f-CGLU are confined within the original information flow of A2C2f. The module still maintains its fundamental components including cv1 feature projection, stacked repeated submodules, feature concatenation and cv2 output fusion. The core adjustment lies in replacing the vanilla MLP branch in ABlock with CGLU when the hyperparameter a2 is set to True. Without disrupting the original feature aggregation pathways, this design introduces local convolutional perception and gated feature screening mechanisms, rendering the model better suited to address the challenges of densely distributed tiny targets, ambiguous boundaries and high inter-class similarity existing in down feather images.

### 3.1. Construction of A2C2f-CGLU Feature Enhancement Module

**Figure 3:**
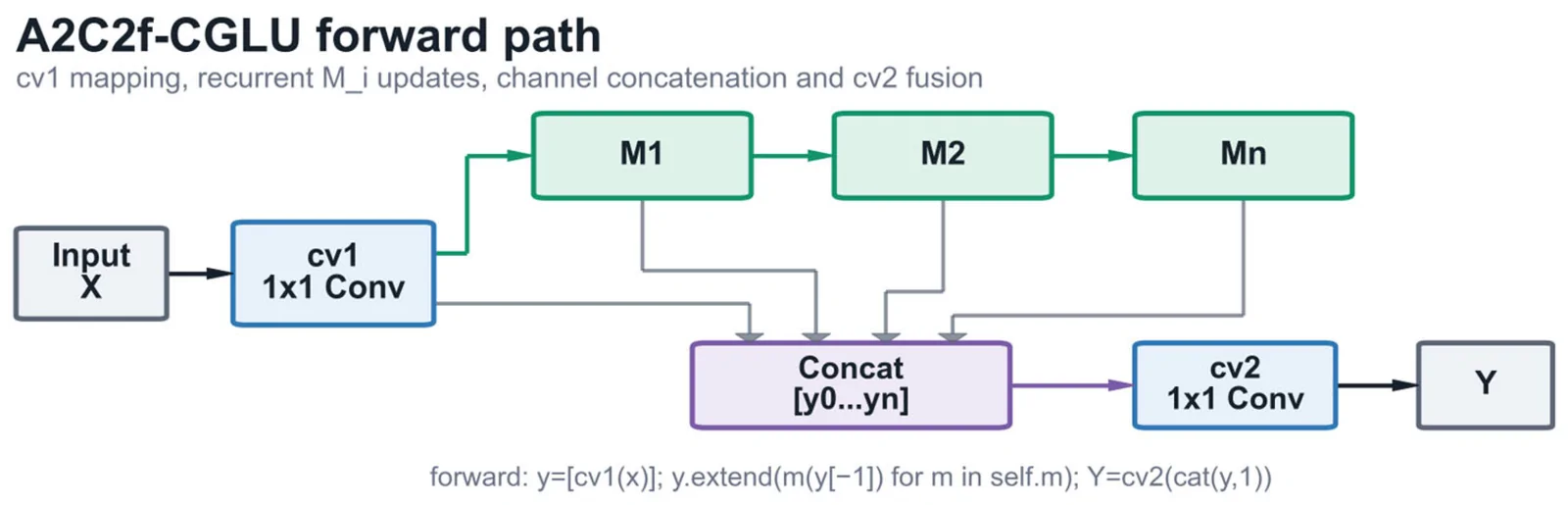
Overall forward feature aggregation pipeline of A2C2f-CGLU.

As illustrated in Figure 3, A2C2f-CGLU inherits the overall feature aggregation pattern of the original A2C2f. The input features are first projected to a hidden channel space via cv1, followed by progressive feature updating through multiple stacked repeated submodules. Finally, output fusion is realized by channel concatenation and cv2. Since the integration of CGLU is confined exclusively inside the intermediate transformation unit, the inherent topology of A2C2f, including input projection, progressive feature propagation and output fusion, remains intact.

To further correspond to the overall forward pipeline illustrated in Figure 3, let the input feature tensor be X, whose dimensional relationship is defined in Equation (1).

(1) $$X\in R^{B\times C_{in}\times H\times W}$$

In Equation (1), B denotes the batch size, $C_{in}$ stands for the number of input channels, and H and W represent the height and width of the feature map, respectively. This input corresponds to the feature tensor received by A2C2f_CGLU.forward(x).

The input features first pass through the entry projection layer ${cv}_{1}$ to be mapped into the hidden channel space, as shown in Equation (2).

(2) $$Y_{0}={cv}_{1}(X),Y_{0}\in R^{B\times C_{e}\times H\times W}$$

In Equation (2), $C_{e}$ denotes the number of hidden channels inside A2C2f. Afterwards, each repeated submodule takes the output from the previous stage as input to implement progressive feature updating, as formulated in Equation (3).

(3) $$Y_{i}=M_{i}(Y_{i-1}),i=1,2,\ldots ,n$$

Equation (3) indicates that the internal features of the module are not simply stacked in parallel, but are transmitted step by step along self.m. Eventually, the features from all stages are concatenated along the channel dimension, and output fusion is implemented via ${cv}_{2}$, as presented in Equation (4).

(4) $$Y_{A2C2f}={cv}_{2}(Concat(Y_{0},Y_{1},\ldots ,Y_{n}))$$

This demonstrates that A2C2f-CGLU retains the original A2C2f pipelines of input projection, progressive updating, channel concatenation and output fusion.

**Figure 4:**
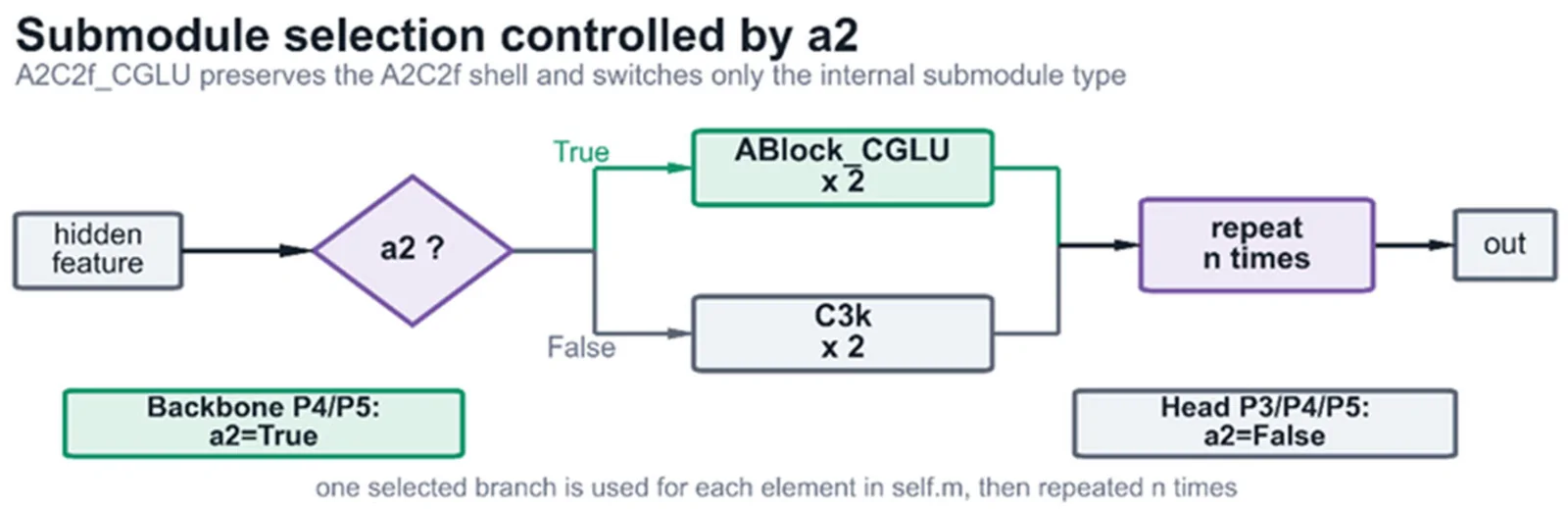
Selection mechanism of repeated submodules driven by the parameter a2.

As illustrated in Figure 4, the parameter a2 inside the module controls the structural form of repeated submodules within self.m. When a2 = True, each repeated unit consists of two sequentially stacked ABlock_CGLU blocks. It captures spatial dependencies via regional attention and strengthens the capacity for local texture modeling with the convolutional gated feed-forward branch. When a2 = False, the repeated unit reverts to the C3k branch without integrating AAttn and CGLU, which reduces extra computational overhead during feature fusion and maintains structural stability. This conditional design avoids indiscriminately introducing complex transformations across all network stages, enabling the model to achieve a more favorable trade-off between feature representation capability and computational efficiency.

The branch selection in Figure 4 is governed by the parameter a2. For the i-th repeated unit in self.m, its structure can be expressed by Equation (5).

(5) $$M_{i}=\{\begin{array}{cc} Seq({ABlock\_CGLU}_{i,1},{ABlock\_CGLU}_{i,2}), & a2=True\\ {C3k}_{i}, & a2=False \end{array}$$

**Figure 5:**
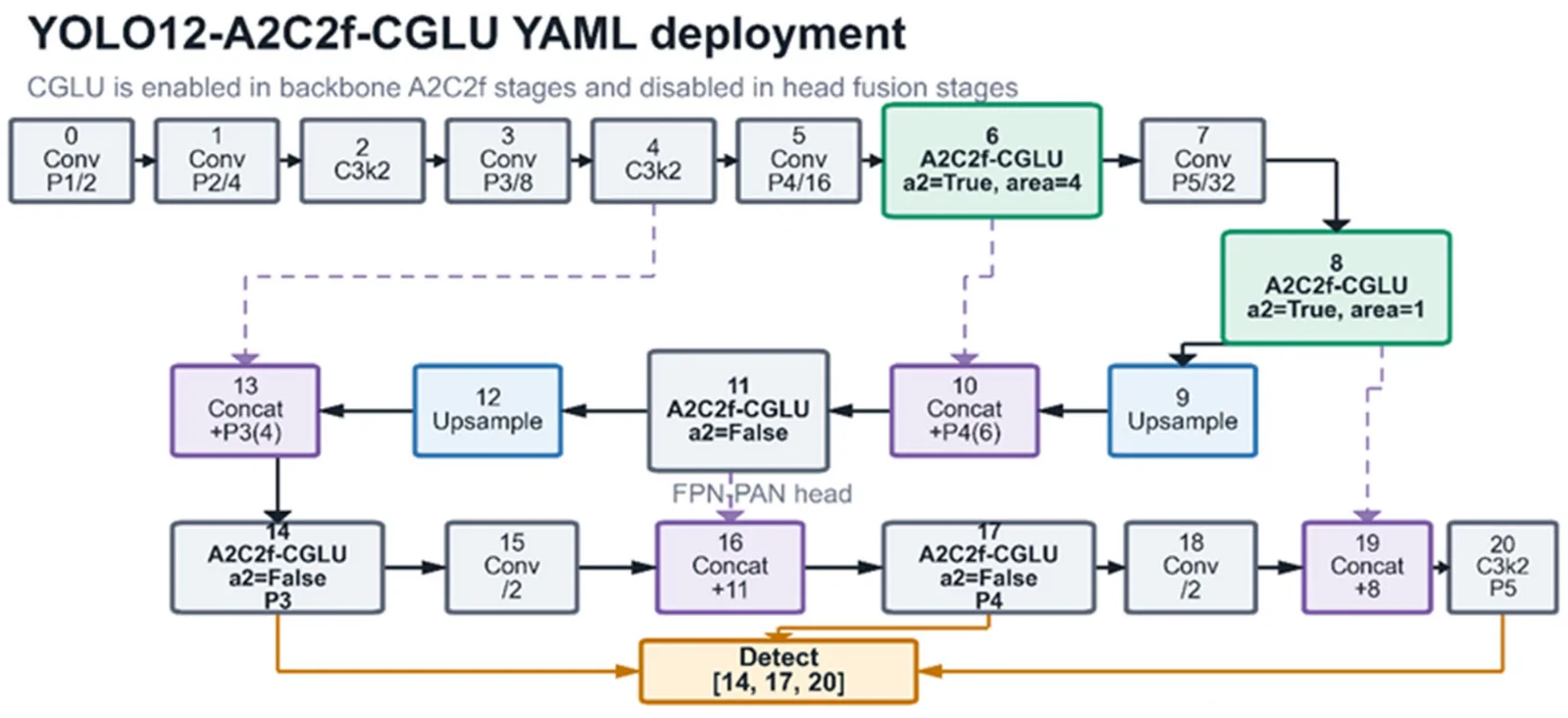
Multi-scale deployment positions of A2C2f-CGLU in YOLO12.

As shown in Figure 5, the YAML example reflects the selective deployment strategy of A2C2f_CGLU. During the deep feature extraction stages P4/P5 of the backbone network, layers 6 and 8 are configured with a2 = True, which forces the repeated submodules to adopt ABlock_CGLU. This setup strengthens regional dependency modeling and local texture filtering at high semantic levels. In contrast, the multi-scale fusion stage of the detection head still employs the A2C2f_CGLU wrapper for layers 11, 14 and 17, yet sets a2 = False for all of them. Their internal repeated units revert to the C3k branch to cut down extra computational overhead along the fusion pathway and stabilize feature propagation. The final detection layers receive multi-scale P3, P4 and P5 features from layers 14, 17 and 20. This configuration embodies the design principle of assigning structural complexity according to functional positions within the network: reinforcing feature representation in deep semantic modeling stages while maintaining lightweight and stable computation in detection head fusion stages.

Figure 5 further illustrates that A2C2f-CGLU is not deployed indiscriminately in the YAML configuration. The layers where CGLU is actually activated are located in the P4/P5 deep feature extraction stages of the backbone, and the set of these layers $L_{CGLU}$ can be formulated as Equation (6).

(6) $$L_{CGLU}=\{6(P4/16,a2=True,area=4),8(P5/32,a2=True,area=1)\}$$

Equation (6) indicates that CGLU is placed in the backbone stages with high semantic levels to boost regional dependency modeling and local texture filtering. Although the A2C2f_CGLU layers in the detection head retain the identical outer module wrapper, they actually adopt the C3k pathway due to the setting a2 = False, as presented in Equation (7).

(7) $$L_{C3k}=\{11,14,17\}$$

Equation (7) corresponds to the lightweight configuration for the P3, P4 and P5 fusion nodes in the detection head. The final detection layers receive multi-scale features from $F_{14}$, $F_{17}$ and $F_{20}$, as given in Equation (8).

(8) $$Y_{det}=Detect(F_{14},F_{17},F_{20})$$

Equation (8) corresponds to the configuration Detect([14,17,20]) in the YAML file, where $Y_{det}$ denotes the final detection output. It demonstrates that the improvements proposed in this work do not alter the original three-scale detection output paradigm of YOLO12.

### 3.2. Attention-Gate Collaborative Mechanism of ABlock_CGLU

**Figure 6:**
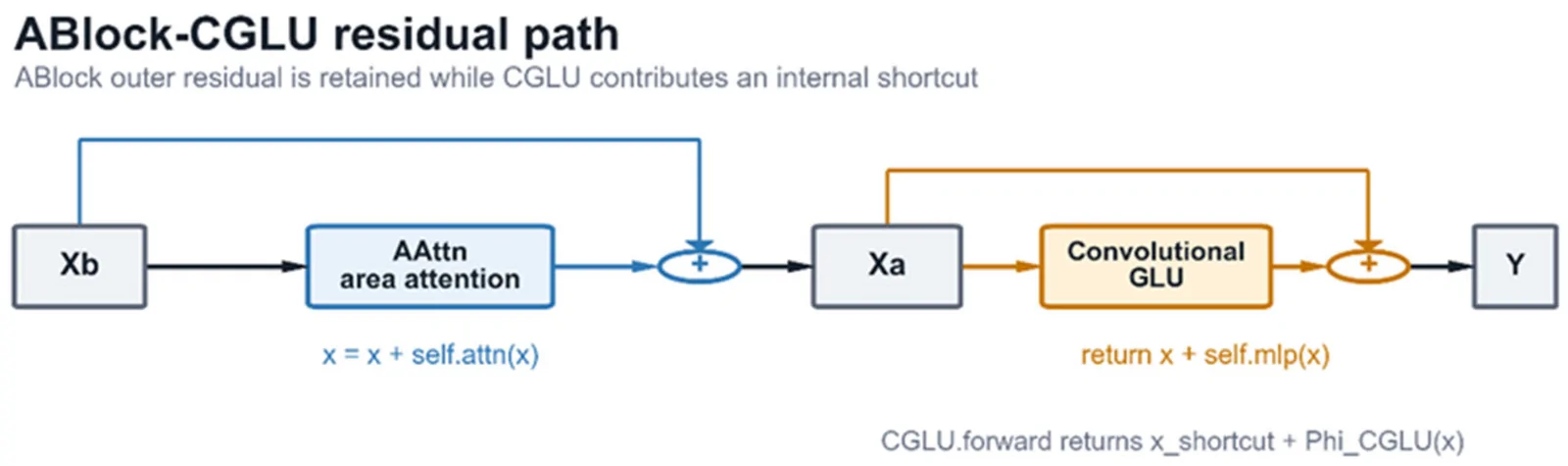
Residual feature updating pipeline of ABlock-CGLU.

ABlock_CGLU inherits the residual organization mode of the original ABlock. The input features first pass through the regional attention branch and perform residual addition with the original input, which enhances valid structural responses by exploiting spatial regional dependencies. Afterwards, the attention-enhanced features are fed into the CGLU feed-forward branch to further extract local texture information and filter channel responses via the gating mechanism. Different from the vanilla ABlock, this work only replaces the ordinary MLP within its feed-forward branch with Convolutional GLU, while keeping the overall computation sequence of “attention residual + feed-forward residual” unchanged. Meanwhile, shortcut connections are preserved inside CGLU, so that the enhanced local textures are superimposed on the input features in a residual manner. Consequently, this design can maintain the stability of original residual propagation and simultaneously improve the model’s capability to model down filaments, edge contours and subtle weak-texture discrepancies, as illustrated in Figure 6.

Figure 6 illustrates the residual updating scheme of ABlock-CGLU. Let the feature fed into a certain ABlock_CGLU be $X_{b}$. The regional attention branch first performs outer residual addition, as expressed in Equation (9).

(9) $$X_{a}=X_{b}+AAttn(X_{b})$$

In Equation (9), AAttn(·) is used to strengthen the dependencies among spatial regions. The attention-enhanced features are then fed into the CGLU feed-forward branch, and a skip connection for input is inherently retained inside CGLU, as shown in Equation (10).

(10) $$G(Z)=CGLU(Z)=Z+\Phi_{CGLU}(Z)$$

In Equation (10), $\Phi_{CGLU}(\cdot )$ denotes the main branch transformation of CGLU. Combined with the feed-forward residual outside ABlock, the output $Y_{B}$ of a single ABlock_CGLU can be formulated as Equation (11).

(11) $$Y_{B}=X_{a}+G(X_{a})=2X_{a}+\Phi_{CGLU}(X_{a})$$

This expression separately represents the outer residual of ABlock and the internal shortcut of CGLU, which can more strictly correspond to the actual forward propagation process in the code.

**Figure 7:**
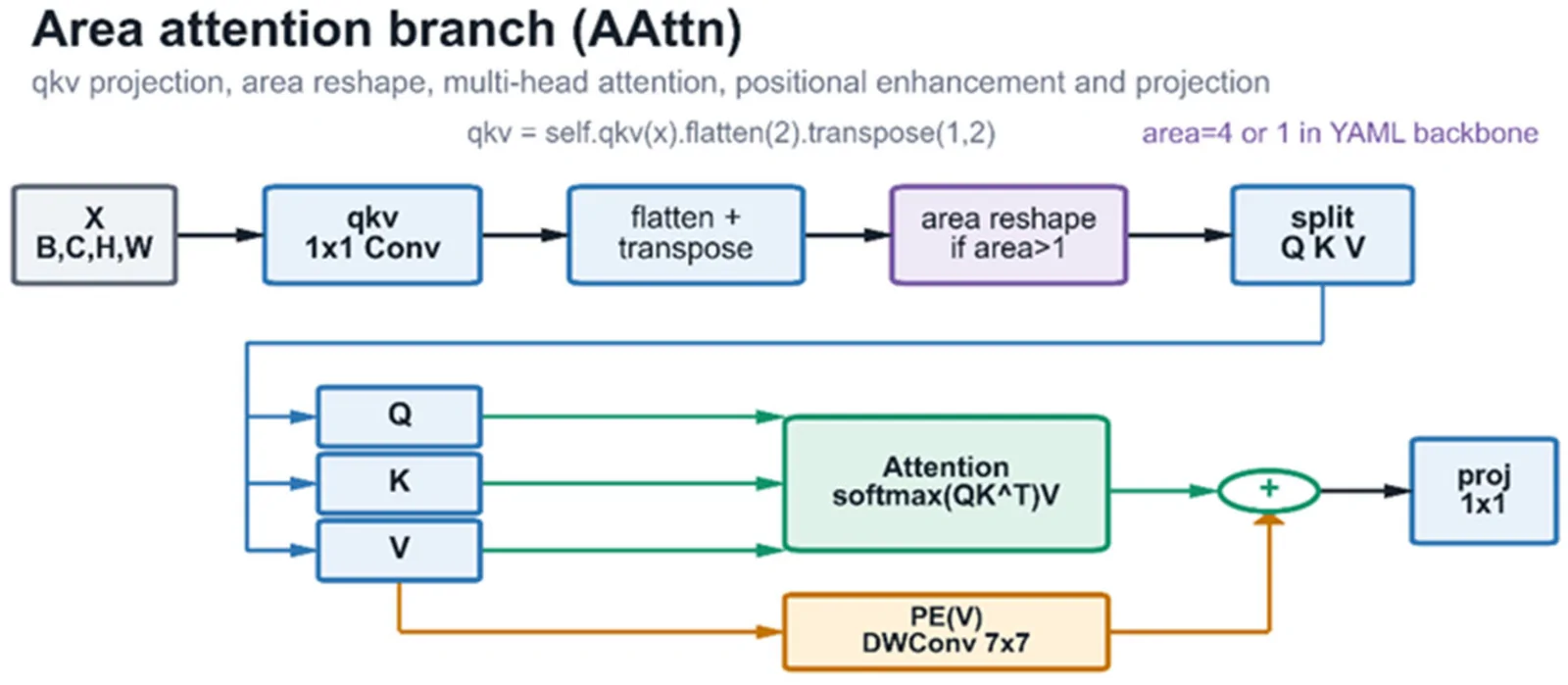
Spatial dependency modeling process of the regional attention branch.

The regional attention branch first maps input features into query, key and value representations via QKV projection. After flattening, transposition and optional regional rearrangement, the features are divided into a format suitable for multi-head attention calculation. Subsequently, the model computes regional attention responses using Q, K and V to build dependencies between different spatial positions. Meanwhile, the position enhancement branch takes V as input, generates PE(V) through depthwise convolution, adds it to the attention output, and applies projection to obtain the final result. For down images featuring loose boundaries, random poses and adjacent tiny down filaments, this branch strengthens valid object responses at the regional level and supplements local spatial bias information. It thus reduces the model’s reliance on the receptive field of a single local convolution, as visualized in Figure 7.

Figure 7 corresponds to the regional attention computation procedure of AAttn. The input features first undergo QKV projection, flattening and transposition, regional rearrangement, and multi-head splitting to form query, key and value tensors, as given in Equation (12).

(12) $$\left[Q,K,V]=Split(Reshape({Conv}_{qkv}(X))\right)$$

The attention response is then calculated from Q, K and V, as shown in Equation (13).

(13) $$O_{attn}=Softmax(\frac{QK^{T}}{\sqrt{d_{h}}})V$$

In Equation (13), $d_{h}$ denotes the channel dimension of a single attention head. To supplement local spatial bias information, the attention output is summed with the position enhancement branch PE(V), followed by projection to produce the final output, as expressed in Equation (14).

(14) $$AAttn(X)=Proj(O_{attn}+PE(V))$$

**Figure 8:**
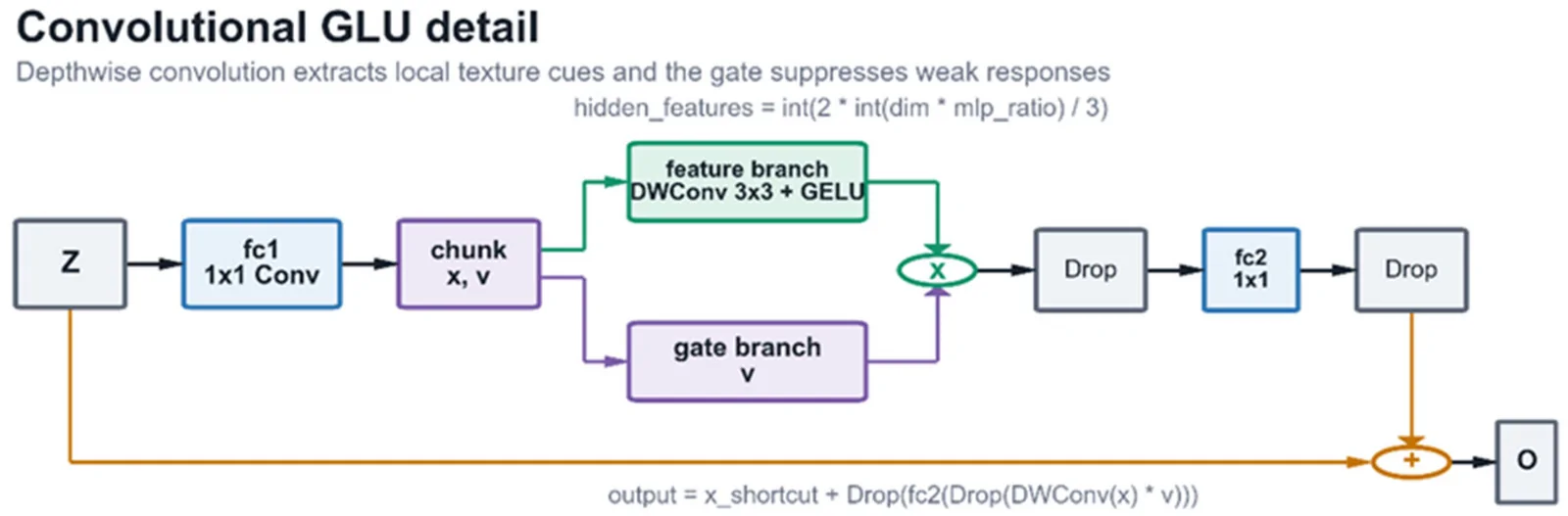
Convolutional gated feature filtering mechanism of the convolutional GLU Branch.

On the basis of retaining the input residual shortcut connection, the CGLU branch reconstructs and filters features from two dimensions: channel mapping and local spatial modeling. Given the input features, the module first expands the hidden representation via a 1 × 1 convolution and splits the feature map along the channel dimension into a feature branch x and a gating branch v. The feature branch passes through a 3 × 3 depthwise convolution and GELU activation to introduce local neighborhood texture information, while the gating branch performs element-wise multiplication with the locally enhanced features to realize content-adaptive response modulation. Subsequently, the fused features sequentially go through dropout, 1 × 1 output projection and another dropout layer, then conduct residual addition with the original input features to obtain the output of CGLU. This process helps strengthen the model’s representation capability for down filaments, cluster edges and subtle weak-texture discrepancies, and suppress interference caused by background fibers, illumination fluctuations and invalid textures, as illustrated in Figure 8.

Figure 8 illustrates the convolutional gated feature filtering process of Convolutional GLU. Firstly, CGLU determines the number of hidden gating channels $C_{g}$ according to the expansion ratio of the MLP in ABlock, as defined in Equation (15).

(15) $$C_{g}=\lfloor \frac{2\lfloor r_{mlp}C_{e}\rfloor }{3}\rfloor$$

Then the input feature Z passes through a 1 × 1 convolution and is split into a feature branch and a gating branch along the channel dimension, as expressed in Equation (16).

(16) $$\left[Z_{f},Z_{g}]={Split}_{c}({Conv}_{1\times 1}(Z)\right)$$

Among them, $Z_{f}$ denotes the feature branch and $Z_{g}$ denotes the gating branch. The feature branch is further processed by a 3 × 3 depthwise convolution and GELU activation to introduce texture information of local neighborhoods, as shown in Equation (17).

(17) $$Z_{f}^{\prime }=GELU({DWConv}_{3\times 3}(Z_{f}))$$

This operation can model local structures such as down filaments, cluster edges and weak textures within each channel. Afterwards, the locally enhanced feature branch $Z_{f}^{\prime }$ and the gating branch $Z_{g}$ perform element-wise multiplication to generate content-adaptive gated responses, as given in Equation (18).

(18) $$Z_{m}=Z_{f}^{\prime }⊙Z_{g}$$

$Z_{m}$ in Equation (18) stands for the gated response. The model can highlight valid texture responses and suppress invalid interference according to input contents. Finally, $Z_{m}$ passes through dropout, a 1 × 1 output projection layer and another dropout layer to produce the main branch output $\Phi_{CGLU}(Z)$ of CGLU, as presented in Equation (19).

(19) $$\Phi_{CGLU}(Z)=Drop({Conv}_{1\times 1}(Drop(Z_{m})))$$

Combined with Equation (10), the final output of CGLU is jointly composed of the input shortcut connection and the convolutional gated main branch. This realizes local texture enhancement and channel response filtering while maintaining residual propagation stability. It should be noted that the base class A2C2f reserves an optional path with a learnable residual scaling factor γ. However, the default parameter residual = False of A2C2f_CGLU is adopted in the YAML configuration of this paper, so this branch is not activated during actual inference and training.

## 4. Experimental Setup

To ensure the reproducibility of experimental results and fair comparisons among different models, all training and validation experiments in this paper are conducted un-der a unified code framework, data split scheme, and hardware and software platform. Except for differences in model architectures, all comparative models adopt identical training protocols and evaluation configurations to mitigate the impacts of varying ex-ternal conditions on experimental outcomes. Each model was trained three times under the same protocol on the same fixed training/validation split, with independent stochastic initializations. The results are reported as the arithmetic mean ± sample standard deviation across the three runs (n = 3 per model). Because runs from different model architectures have no natural one-to-one correspondence, two-sided Welch two-sample *t*-tests were used to compare each baseline model with A2C2f-CGLU (α = 0.05).

The operating system of the experimental platform is Microsoft Windows 11 Pro 64-bit with build version 10.0.26200. For hardware specifications: the CPU is Intel(R) Core(TM) Ultra 7 270K Plus, equipped with 24 GB system RAM. The graphics card is NVIDIA GeForce RTX 5080 with 16 GB video memory and driver version 581.15. The deep learning environment is built with Python 3.11.15, PyTorch 2.5.0 + cu118, Torchvision 0.20.0 + cu118 and Ultralytics 8.3.9. Auxiliary dependency libraries include OpenCV 4.13.0, NumPy 2.4.6, Pandas 3.0.2 and timm 1.0.27. All training and validation procedures are assigned to device = 0 and executed on a single GPU.

The experimental dataset follows the YOLO object detection annotation format. The dataset consists of 1140 down images in total, which are split into a training set and a validation set at an approximate ratio of 8:2. Specifically, the training set contains 911 images and the validation set contains 229 images. Since no independent test subset is configured in the experimental setup, the model performance evaluation relies on metrics derived from the validation set. Five detection categories are defined: yellow_feather, down_silk, immature_down, feather, and down. These correspond to fine-grained components that need to be distinguished in down quality assessment, namely discolored feathers, down filaments, immature down, feathers, and pure down, respectively.

The total training epoch is set to 100 with a batch size of 16. All input images are uniformly resized to a resolution of 640 × 640, and the number of data loading workers is configured as four. The optimizer is automatically selected by the Ultralytics framework. The initial learning rate lr0 is set to 0.01, the final learning rate scaling factor lrf is 0.01, the momentum coefficient is 0.937, and the weight decay coefficient is 0.0005. A cosine learning rate scheduler is adopted throughout training, and warmup_epochs is set to 3.0 to mitigate the negative impact of gradient oscillation in the early training stage on the learning of fine-grained texture features.

In terms of loss function weights, the bounding box regression loss box is set to 7.5, the classification loss cls to 0.5, and the Distributed Focal Loss (DFL) dfl to 1.5. To boost training stability and generalization performance, mixed precision training is enabled by setting amp = True, alongside dropout = 0.1 and label_smoothing = 0.05. Since this paper focuses on performance discrepancies brought by different structural improvements under identical training pipelines, the early stopping strategy is disabled with patience = 0, and all models are fully trained for the predefined number of epochs.

Considering the characteristics of down images, such as tiny object scales, random poses, loose boundaries and severe interference from background fibers, a comprehensive data augmentation pipeline is adopted during training. Geometric augmentations include random rotation (degrees = 10.0), translation (translate = 0.1), scaling (scale = 0.5), shearing (shear = 5.0) and perspective transformation (perspective = 0.0005), with the horizontal flip probability set to fliplr = 0.5. Color space augmentations are configured as hsv_h = 0.05, hsv_s = 0.7 and hsv_v = 0.6 to simulate color variations under diverse illumination and shooting conditions. Composite sample augmentations consist of mosaic (mosaic = 1.0), mixup (mixup = 0.4) and copy–paste (copy_paste = 0.3). Random augmentation and random erasing (erasing = 0.3) are also applied to strengthen the model’s adaptability to occlusion, local feature loss and complex background noise. The mosaic augmentation is disabled in the final 10 epochs. This operation guides the model to gradually fit the distribution of real images in the late training phase and improves convergence stability.

## 5. Experimental Results

To verify the effectiveness of the proposed A2C2f-CGLU architecture for fine-grained down object detection, the original YOLO12 is taken as the baseline model. Two additional improved variants, A2C2f-DFFN and A2C2f-KAN, are introduced for horizontal comparison. DFFN and KAN are feed-forward transformation and nonlinear modeling structures widely adopted in recent years to boost feature representation capacity. Including them in comparative experiments helps distinguish whether the performance gain of A2C2f-CGLU stems from the targeted modeling of local textures, weak boundaries and inter-class subtle differences among down components by CGLU, rather than a simple rise in structural complexity brought by feed-forward module replacement. With identical data partitioning, training schemes and evaluation metrics, this paper systematically compares all models from multiple dimensions: overall detection performance, training dynamics, loss convergence, per-class PR curves, normalized confusion matrices, and visualization results on real test samples. The experimental design not only focuses on the improvement of average precision, but also further investigates model behaviors including category confusion, convergence stability and practical detection usability. This enables a comprehensive evaluation of the applicability of A2C2f-CGLU to the real-world down quality identification scenario. The mean ± SD values and corresponding statistical comparisons based on three independent runs are summarized in Table 1, with *p*-values calculated using two-sided Welch two-sample *t*-tests.

For consistency with the original experimental design and the visual analyses, all subsequent detailed analyses of training dynamics, PR curves, confusion matrices and representative detection results are based on the first experimental run (Run 1). All three independent runs were used to quantify run-to-run variability by calculating the mean and sample standard deviation and to perform the corresponding statistical comparisons.

**Figure 9:**
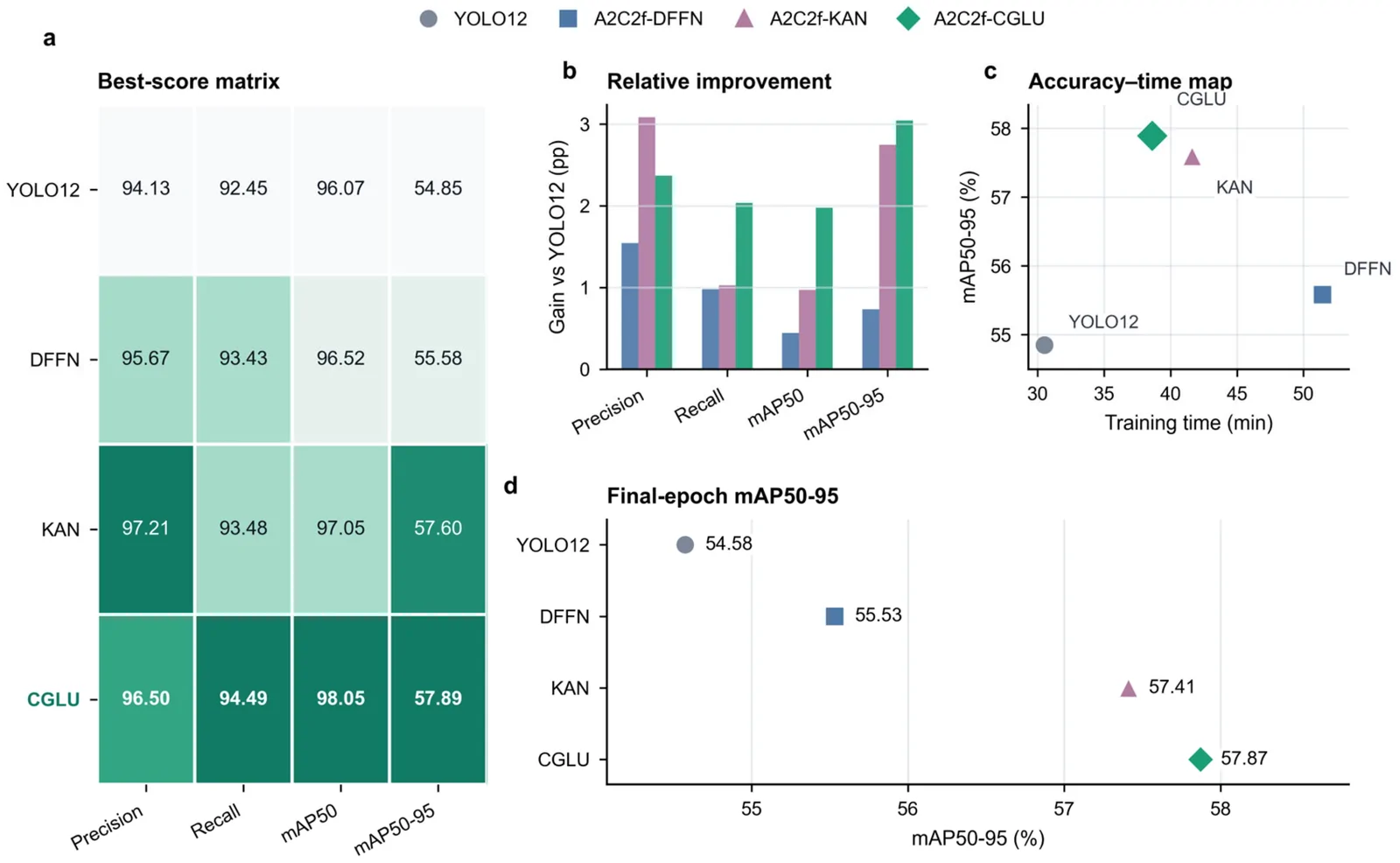
Comparison of overall detection performance and efficiency.

Figure 9 compares the comprehensive performance of different models from four dimensions: overall detection accuracy, relative performance gain, training time, and stability at the final training epoch.

Figure 9a presents the precision, recall, mAP50 and mAP50-95 of each model at the epoch with optimal mAP50-95. The proposed A2C2f-CGLU achieves recall of 94.49%, mAP50 of 98.05% and mAP50-95 of 57.89%, which demonstrates its more stable recall and localization capacity for tiny objects and weakly bounded targets in down images.

Figure 9b further illustrates that A2C2f-CGLU yields a 3.04 percentage point improvement in mAP50-95 compared with the original YOLO12, accompanied by obvious gains in recall and mAP50. This indicates that the convolutional gated branch delivers coordinated improvements in detection completeness and average precision, rather than only boosting a single metric.

As shown in Figure 9c, CGLU achieves the highest mAP50-95 with less training time than DFFN, showing a favorable accuracy–efficiency trade-off.

In Figure 9d, A2C2f-CGLU still retains a mAP50-95 of 57.87% at the final epoch. This proves that its performance superiority does not rely on a single accidental optimal checkpoint, and the model possesses strong stability in the late training phase.

For down quality identification tasks, this result implies that the model can reliably support subsequent component proportion statistics and quality grade evaluation.

**Figure 10:**
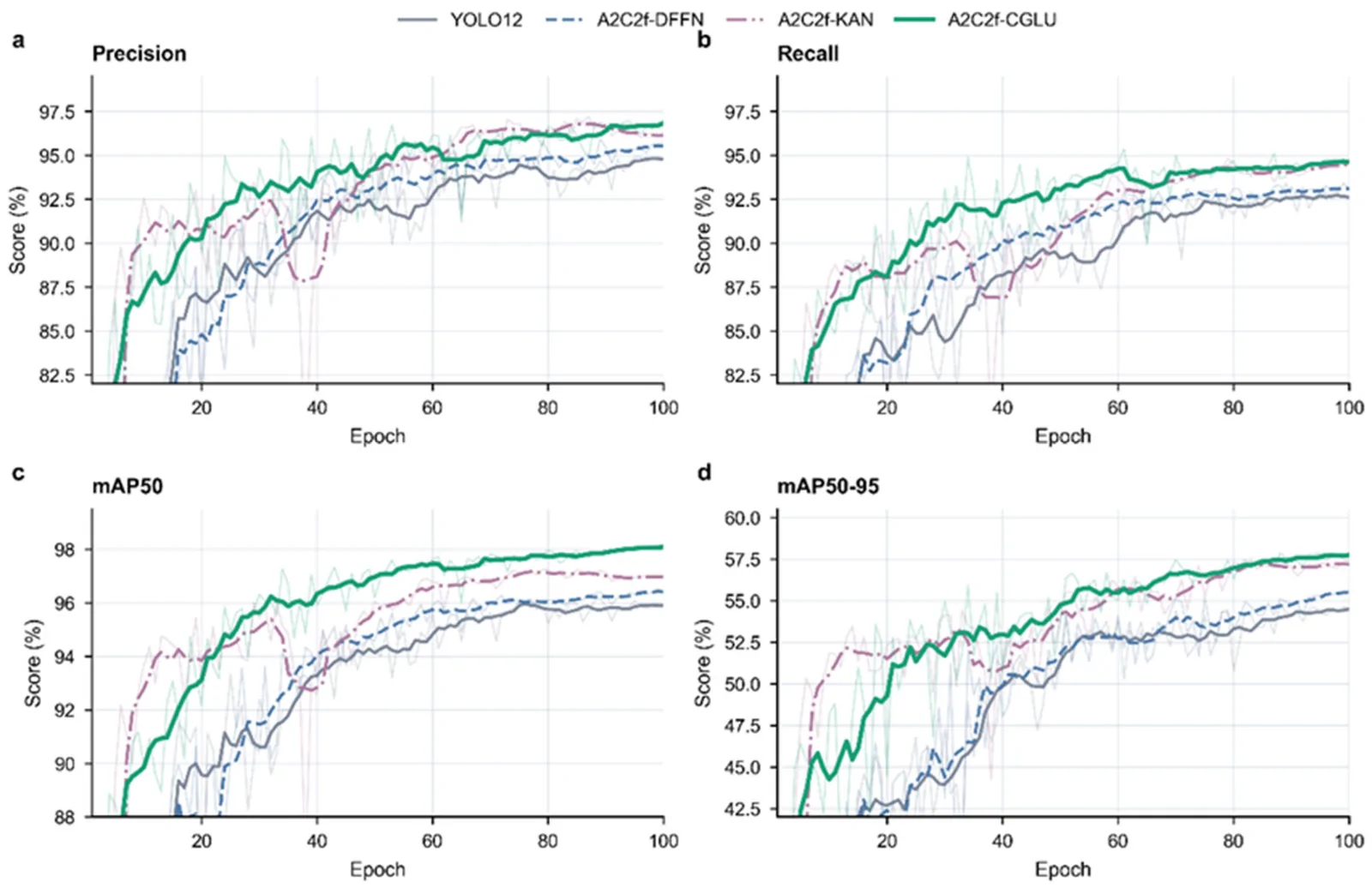
Variation in detection metrics during training.

Figure 10 depicts the variation curves of precision, recall, mAP50 and mAP50-95 of the four models over 100 training epochs to analyze how performance gains are formed during training.

In Figure 10a, the precision of A2C2f-CGLU keeps rising in the middle and late training stages and remains at a high level at the end, revealing that the model’s ability to suppress false detections gradually strengthens as training proceeds.

Figure 10b shows that the recall of the CGLU variant maintains advantages in both the early and mid-to-late training phases, which verifies that the convolutional gated structure helps reduce missed detections of tiny and densely distributed down targets.

In Figure 10c, the mAP50 curve of CGLU is consistently higher than those of other models, demonstrating its more stable overall performance in category recognition and object localization under a loose IoU threshold.

Figure 10d further indicates that CGLU maintains a leading position in the late training stage even under the stricter mAP50-95 metric, which is particularly critical for down targets with loose boundaries and irregular shapes.

Overall, the superiority of A2C2f-CGLU persists throughout the entire training process rather than only emerging in the final epoch. This indicates that the model achieves more sufficient learning of down textures, object edges, and subtle inter-class distinctions among similar down components.

**Figure 11:**
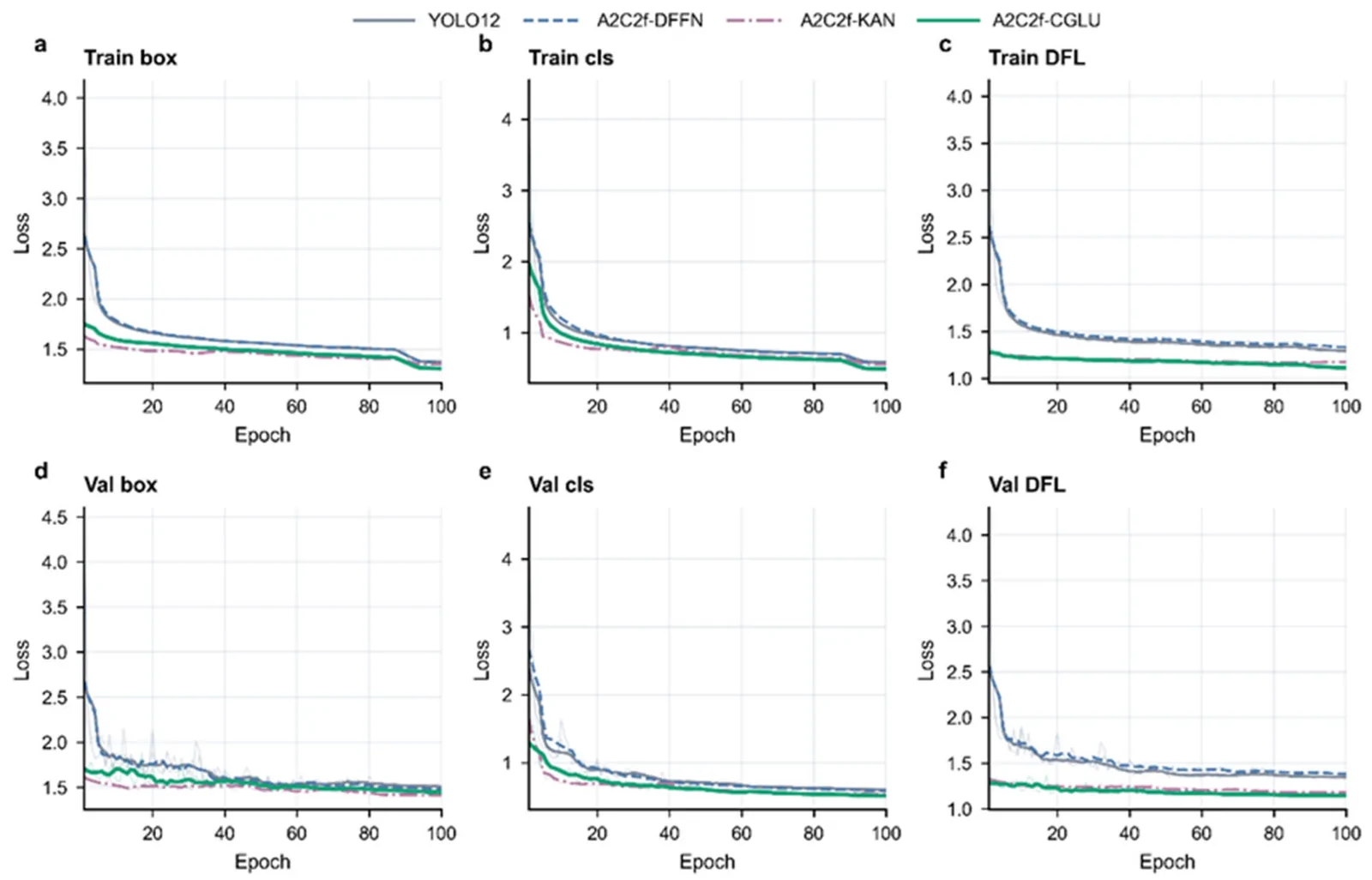
Convergence behavior of loss functions.

Figure 11 analyzes the optimization stability of different models through box loss, cls loss and DFL on the training set and validation set.

Figure 11a,d illustrate the bounding box regression loss of the training set and validation set respectively. The box loss of A2C2f-CGLU declines steadily in the late training stage, proving that the introduction of CGLU does not impair the original localization optimization pipeline of YOLO12.

Figure 11b,e show a continuous drop in classification loss, which implies that the model establishes more robust classification decision boundaries among highly similar components including yellow_feather, down_silk, immature_down, feather and down.

In Figure 11c,f, the DFL of the CGLU variant stays at a lower level with mild fluctuations, reflecting more stable learning for the boundary distribution of bounding boxes.

Down targets generally suffer from blurred contours, scattered fibers and partial overlap. Steady box loss and DFL contribute to higher bounding box localization quality, while reduced cls loss alleviates category confusion among down filaments, immature down and feathers.

From the perspective of optimization dynamics, these results validate the rationality of the A2C2f-CGLU design, which achieves feature enhancement while preserving stable residual training.

**Figure 12:**
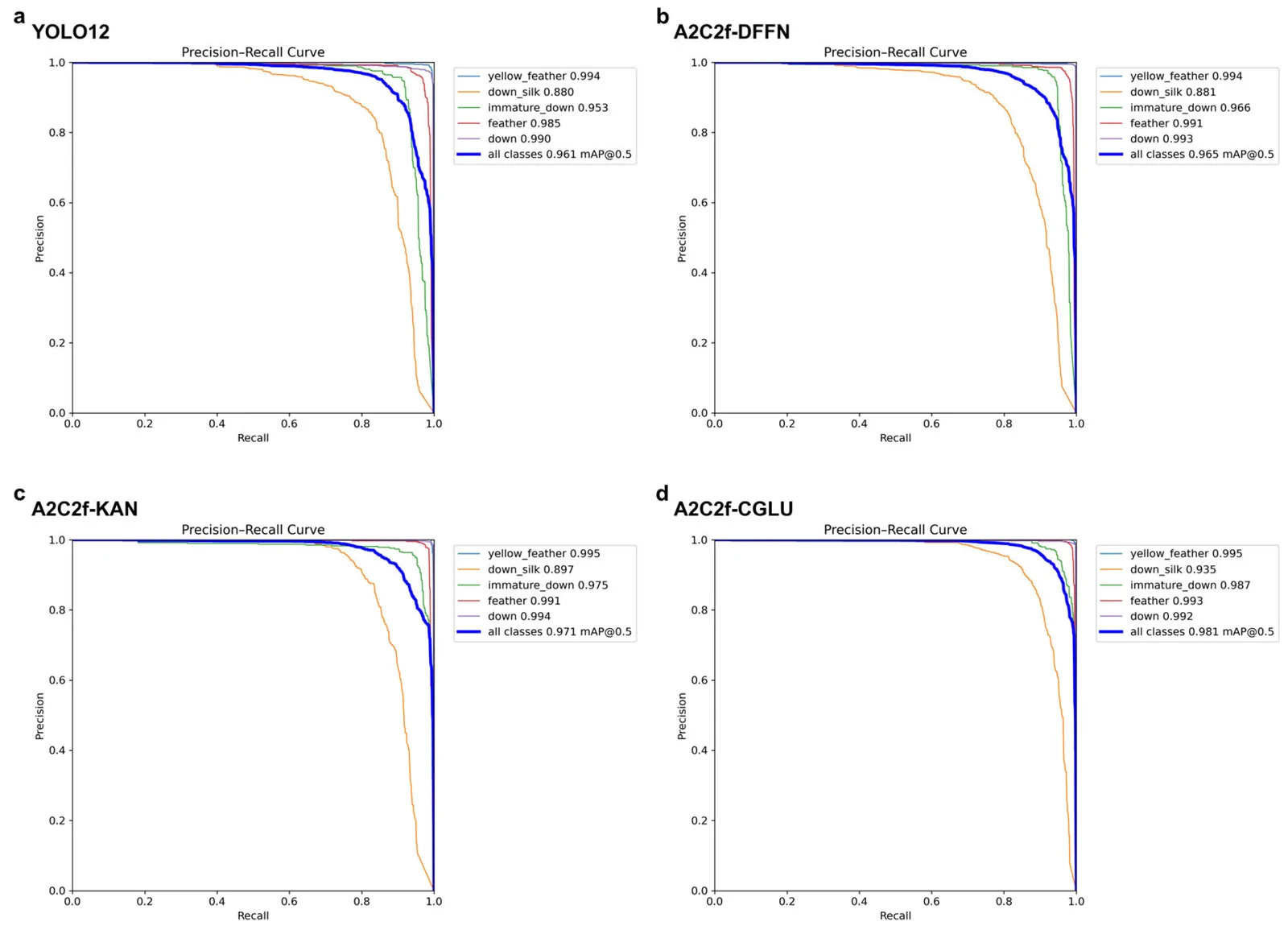
Per-class precision–recall curves.

Figure 12 adopts per-class precision–recall (PR) curves to further assess the precision-recall trade-off of different models on each down component.

As illustrated in Figure 12a, the original YOLO12 achieves an overall mAP@0.5 of 0.961, while the AP of down_silk only reaches 0.880. This indicates that down filaments, characterized by tiny scales, scattered shapes and frequent confusion with background fibers, constitute one of the primary challenges in this detection task.

In Figure 12b, the overall mAP@0.5 of A2C2f-DFFN rises to 0.965, yet the AP of down_silk merely improves to 0.881 with marginal gains.

Figure 12c shows that A2C2f-KAN lifts the overall mAP@0.5 to 0.971 and optimizes the curve profiles of categories such as immature_down.

In Figure 12d, the proposed A2C2f-CGLU attains an overall mAP@0.5 of 0.981. The AP of down_silk is greatly boosted to 0.935, and immature_down, feather and down also maintain high AP values.

These results demonstrate that CGLU not only elevates the overall mean detection precision but, more crucially, enhances the model’s ability to retain high precision within high-recall intervals for filamentous, small-scale and weak-texture categories. This performance characteristic aligns closely with the practical demands of down quality inspection, which requires the stable identification of scarce, tiny and easily confused down constituents.

**Figure 13:**
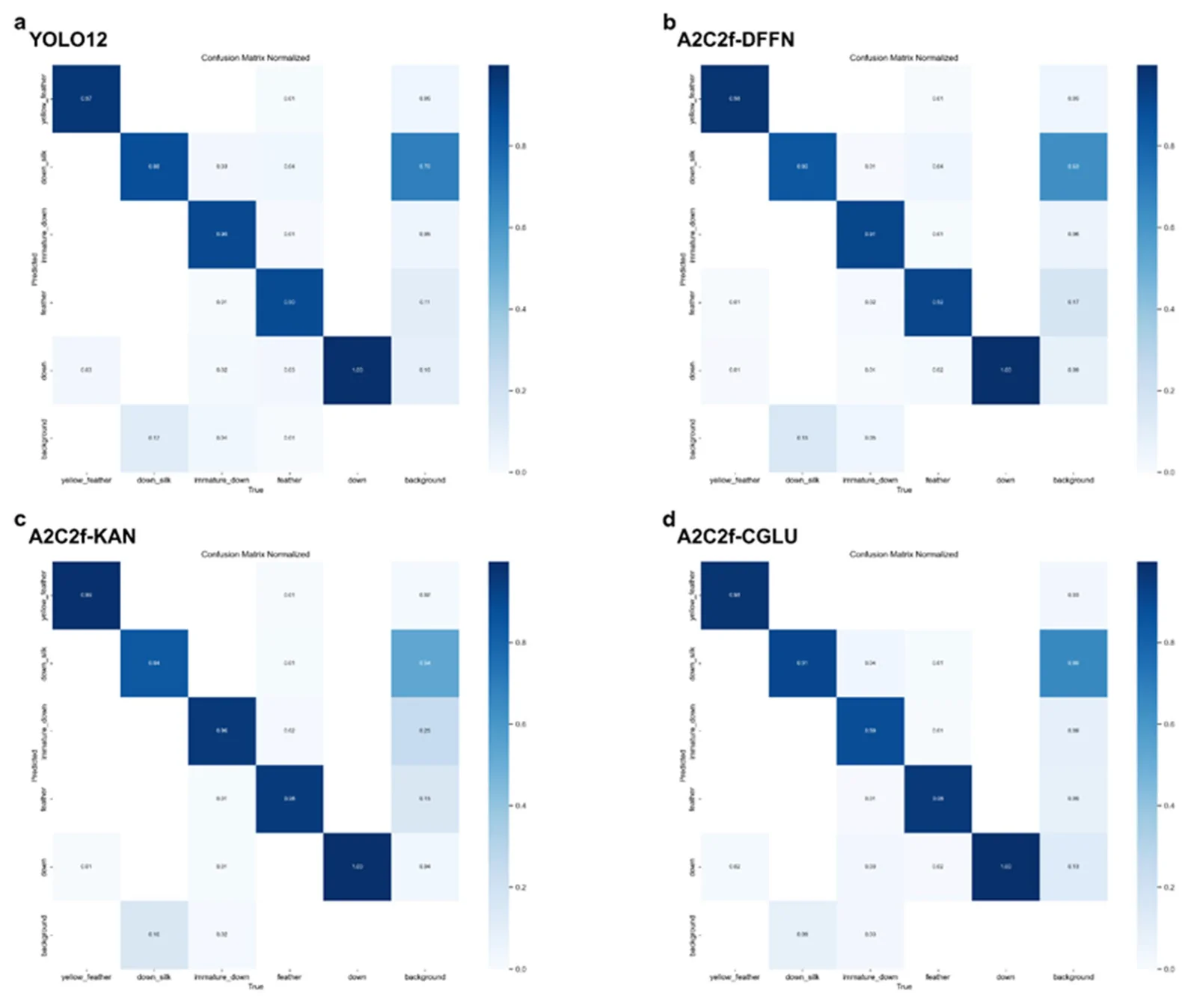
Normalized confusion matrices.

Figure 13 analyzes the class discrimination capacity of different models from the perspective of error distribution via normalized confusion matrices.

As shown in Figure 13a, the original YOLO12 performs well on categories with distinct visual features such as down and yellow_feather. Nevertheless, the diagonal values corresponding to down_silk and feather are relatively low, which indicates that the model frequently confuses slender fibrous structures and feather-like analogical shapes.

Figure 13b reveals that A2C2f-DFFN delivers moderate improvements for yellow_feather and immature_down, yet the proportion of correct predictions for down_silk remains unsatisfactory with marginal promotion.

In Figure 13c, A2C2f-KAN achieves clearer separation for immature_down, but the detection accuracy of down_silk still lacks obvious enhancement.

Figure 13d demonstrates that A2C2f-CGLU attains a diagonal value of 0.91 for down_silk and 0.96 for feather, while the down category maintains a perfect stable recognition rate of 1.00. This verifies that the convolutional gated structure possesses superior screening capability for categories heavily dependent on local textures, including down filaments and feathers.

It is worth noting that residual inter-class confusion still exists for immature_down, which implies that the fine-grained visual boundary between immature down and other downy constituents remains a core direction for subsequent optimization.

From the perspective of error types, this figure illustrates that the performance gain of A2C2f-CGLU mainly stems from reduced classification uncertainty among the most easily confused critical categories.

**Figure 14:**
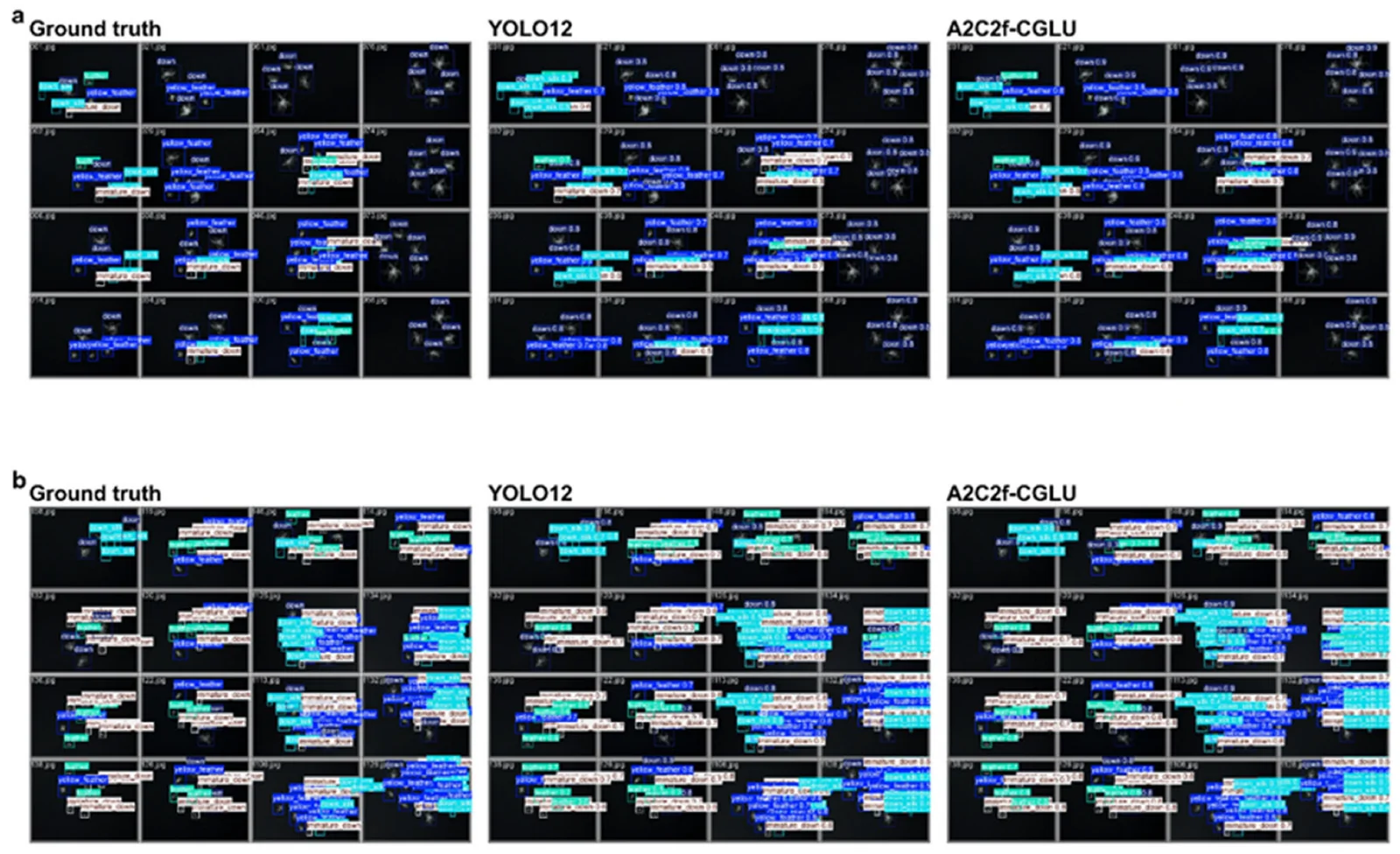
Representative detection results on real down-feather validation images. (**a**) Detection results for a relatively sparse sample with a clean background; from left to right, the panels show ground-truth annotations, YOLO12 predictions and A2C2f-CGLU predictions. (**b**) Detection results for a complex sample with densely distributed objects and multiple coexisting categories; the panels are arranged in the same order.

Figure 14 presents the detection comparison results on real validation samples to verify whether quantitative metrics can be converted into effective detection performance on practical images.

Figure 14a displays a sample with relatively sparse objects and a clean background. Compared with the original YOLO12, A2C2f-CGLU generates more adequate responses for tiny down_silk, partially overlapped down clusters and feather targets with irregular edges, and its predicted bounding boxes align better with manual annotations.

Figure 14b illustrates a complex sample featuring densely packed objects and coexisting multiple categories. In such scenarios, the original YOLO12 is prone to adjacent target adhesion, partial missed detections and insufficient classification confidence. By contrast, A2C2f-CGLU produces more consistent detection outputs for similar categories including down filaments, immature down and feathers.

These visualization results prove that the advantages of CGLU are not merely reflected in improved quantitative metrics, but also embodied in strengthened recognition stability for weak-boundary, small-scale and densely arranged targets on real down images. For subsequent down quality grading tasks, more complete instance detection results can boost the reliability of component counting and proportion estimation.

## 6. Conclusions and Future Work

Aiming at the limitations of manual down quality inspection, including low sorting efficiency, strong subjectivity and difficulty in identifying fine-grained down constituents, this paper proposes a YOLO12-based fine-grained component detection method enhanced by the A2C2f-CGLU feature module. The proposed model targets five annotated categories, namely yellow_feather, down_silk, immature_down, feather and down. In the running text, these categories correspond to discolored feathers, down filaments, immature down, feathers and pure down, respectively. By replacing the vanilla MLP feed-forward branch in ABlock with ConvolutionalGLU, the proposed method introduces local convolutional perception and gated feature selection while preserving the original regional attention and residual propagation structure of A2C2f. This design strengthens the representation of filamentous structures, clustered down edges, weak texture variations and ambiguous boundaries among visually similar components.

Experimental results show that A2C2f-CGLU achieves competitive performance in recall, mAP50 and mAP50-95 under the current validation protocol. Specifically, the mAP50-95 reaches 57.89%, representing a 3.04 percentage point improvement over the original YOLO12 baseline. In addition, PR curves, normalized confusion matrices and detection visualizations indicate that the proposed structure improves the recognition stability of small, weak-boundary and texture-dependent down components. These results support the effectiveness of A2C2f-CGLU as a component-level visual detection module for down quality assessment.

From an application perspective, the proposed method does not directly output final commercial quality grades. Instead, it provides category-level detection results for subsequent component counting, proportion calculation and quality-grade judgment. Since down quality evaluation relies on the category, quantity and proportion of different constituents, the ability to distinguish pure down, down filaments, immature down, feathers and discolored feathers provides an important visual basis for automated, standardized and traceable down quality assessment.

Despite the above findings, several limitations remain and should be addressed in future work.

(1) The current dataset was collected from a single down processing scenario, with limited sample size and imaging variation. Therefore, although the experiments provide controlled evidence for comparing different model structures, the cross-scene generalization ability of the proposed method still requires further validation. Future work will expand the dataset using samples from different producing areas, production batches, acquisition dates, illumination conditions, camera devices and factory environments.
(2) The present evaluation is based on 911 training images and 229 validation images, without an independent test subset. The optimal checkpoint is selected according to validation mAP50-95 and then evaluated on the same validation set. Thus, the reported results should be interpreted as comparative validation performance under the current experimental protocol, rather than unbiased estimates on an untouched test set. Future studies will construct independent training, validation and test subsets, and will further introduce external industrial inspection data to evaluate model stability under practical production conditions.
(3) The repeated-run analysis included only three independent training runs per model (n = 3) under a fixed training/validation partition. Although the mean ± SD values characterize run-to-run variability and the two-sided Welch two-sample *t*-tests provide preliminary between-model comparisons, the small number of runs limits statistical power, while the fixed data partition restricts the generalizability of the inferential results. Future work will include more random seeds, multiple independent training/validation splits, an independent test set, and external industrial inspection data.
(4) In the current implementation, A2C2f-CGLU preserves the outer residual organization of ABlock, while ConvolutionalGLU also contains an internal shortcut connection. Therefore, the reported improvement should be understood as the effect of the complete A2C2f-CGLU configuration with nested residual propagation. Since this study does not include a dedicated ablation experiment separating the outer residual connection, the internal CGLU shortcut and the convolutional gating operation, their individual contributions remain to be further quantified.
(5) Although A2C2f-CGLU improves the recognition of several fine-grained categories, residual confusion still exists among visually analogous targets, especially immature down and other down-like components. Future studies can further improve the discriminative ability for highly similar categories by refining annotation standards, increasing difficult samples, introducing class re-sampling strategies and designing multi-scale texture enhancement mechanisms.

Future research will also focus on model lightweighting and practical system deployment. On the one hand, the parameter size and inference cost of the model can be further reduced while preserving detection accuracy, making it more suitable for edge devices on production lines or online inspection terminals. On the other hand, the detection outputs can be integrated with component proportion calculation, quality-grade judgment and inspection report generation modules, thereby forming a complete intelligent inspection pipeline covering image acquisition, target recognition and quantitative quality assessment.

## Figures and Tables

**Table 1:** Mean ± SD and Welch two-sample *t*-test results.

Model	Analysis	Precision (%)	Recall (%)	mAP50 (%)	mAP50-95 (%)
YOLO12	Mean ± SD	94.93 ± 0.71	92.85 ± 0.46	96.20 ± 0.26	54.77 ± 0.07
A2C2f-DFFN	Mean ± SD	95.72 ± 0.07	93.34 ± 0.08	96.59 ± 0.08	55.47 ± 0.21
A2C2f-KAN	Mean ± SD	96.84 ± 0.70	93.55 ± 0.78	96.95 ± 0.49	56.84 ± 0.71
A2C2f-CGLU	Mean ± SD	97.14 ± 0.58	95.92 ± 1.24	98.13 ± 0.07	59.07 ± 1.03
YOLO12 vs. CGLU	*p*-value	0.0150	0.0374	0.0038	0.0181
A2C2f-DFFN vs. CGLU	*p*-value	0.0495	0.0686	<0.0001	0.0224
A2C2f-KAN vs. CGLU	*p*-value	0.5963	0.0593	0.0512	0.0427

Note: Values are reported as percentages. Mean ± SD denotes the arithmetic mean ± sample standard deviation across three independent training runs (n = 3 per model). *p*-values were calculated using two-sided Welch two-sample *t*-tests, treating the three runs for each model as independent observations and comparing each baseline model with A2C2f-CGLU (α = 0.05); no pairing by run index was assumed.

## Data Availability

The data used in this study are available from the corresponding author upon reasonable request.
